# Paradoxical reaction in tuberculous meningitis: presentation, predictors and impact on prognosis

**DOI:** 10.1186/s12879-016-1625-9

**Published:** 2016-06-21

**Authors:** Anurag Kumar Singh, Hardeep Singh Malhotra, Ravindra Kumar Garg, Amita Jain, Neeraj Kumar, Neera Kohli, Rajesh Verma, Praveen Kumar Sharma

**Affiliations:** Department of Neurology, King George Medical University, Uttar Pradesh Lucknow, PIN-226003 India; Department of Microbiology, King George Medical University, Uttar Pradesh Lucknow, India; Department of Radiodiagnosis, King George Medical University, Uttar Pradesh Lucknow, India

**Keywords:** Tuberculoma, Optochiasmatic arachnoiditis, Hydrocephalus, Cerebrospinal fluid, Immune reconstitution inflammatory syndrome

## Abstract

**Background:**

Awareness about paradoxical reactions in tuberculous meningitis is crucial as a paradoxical reaction may lead to certain wrong conclusions (for example, an erroneous diagnosis, and a possibility of treatment failure, mycobacterial drug-resistance, drug toxicity, or presence of a malignancy). The present study was planned to evaluate the incidence and predictive factors of paradoxical reactions in light of clinical, cerebrospinal fluid, and neuroimaging characteristics.

**Methods:**

In this prospective cohort study, consecutive patients fulfilling the International Consensus criteria of tuberculous meningitis were included. Patients were subjected to clinical evaluation, cerebrospinal fluid evaluation, and neuroimaging. Patients were treated with anti-tuberculosis drugs along with corticosteroids. Patients were regularly followed up at 3 monthly intervals. At each follow up patients were evaluated clinically and repeat cerebrospinal fluid analysis was performed along with repeat neuroimaging. Disability assessment was done using Barthel index.

**Results:**

We enrolled 141 patients of tuberculous meningitis. Approximately one-third of patients (44/141; 31.2 %) developed a paradoxical reaction. Twenty-seven patients developed hydrocephalus, 26 developed tuberculomas, 12 developed optochiasmatic arachnoiditis and 4 patients had spinal arachnoiditis. In 41 patients (out of 44) cerebrospinal fluid paradoxically worsened (increase in cells and/or protein); 2 demonstrated a decrease in cells with polymorph predominance while in one it was normal. In 3 patients, paradoxical cerebrospinal fluid changes were not associated with neuroimaging changes. On multivariate analysis, predictors of paradoxical reaction were female gender (*p* = 0.013), HIV positivity (*p* = 0.01) and a shorter duration of illness (*p* = 0.049). Development of paradoxical reactions did not predict the disability status of the patients.

**Conclusions:**

Paradoxical reaction occurs in approximately one-third of patients with tuberculous meningitis. Female gender, concomitant HIV infection, and a shorter duration of illness were significant predictors. Paradoxical reactions did not adversely affect the outcome.

**Electronic supplementary material:**

The online version of this article (doi:10.1186/s12879-016-1625-9) contains supplementary material, which is available to authorized users.

## Background

Paradoxical reaction, in patients with tuberculous meningitis, is characterized by either worsening of pre-existing tuberculous lesions or the appearance of new tuberculous lesions in patients who show initial improvement following anti-tuberculosis treatment. The clinical or radiological deterioration associated with such a reaction may fallaciously suggest either a drug resistant state or treatment failure, and might even prompt to look for an alternative diagnosis [1–3]. The manifestations of paradoxical reaction, that have been described, are mostly derived from case reports and small case series. These include a variety of clinical manifestations and neuroimaging abnormalities, along with an altered cerebrospinal fluid picture. Cerebrospinal fluid changes may include lymphocytic pleocytosis, a change in cellular components in the direction of polymorphonuclear predominance, or an increase in protein levels. Paradoxical reaction does not necessarily represent treatment failure and corticosteroids have been shown to have a beneficial effect [4–6].

In human immunodeficiency virus (HIV)-infected patients, paradoxical reactions are termed as the immune reconstitution inflammatory syndrome (IRIS), which is a condition characterized with worsening of pre-existing infective processes, following treatment with antiretroviral therapy. Tuberculosis-associated immune reconstitution inflammatory syndrome is characterized by worsening of clinical symptoms, signs, and imaging characteristics of tuberculosis subsequent to initiation of antiretroviral therapy after initial improvement with anti-tuberculosis treatment [7, 8].

Most of the current information about paradoxical reaction in tuberculous meningitis is in the form of isolated case reports, small case series, and articles with small number of patients. The exact frequency and spectrum of paradoxical reaction is not precisely known. Its impact on prognosis is also not clear. This prospective follow up study has been planned to answer all these questions.

## Methods

This prospective study was conducted in Department of Neurology, King George Medical University Uttar Pradesh, Lucknow. Our institute is a tertiary care hospital serving a population of approximately 100 million. It is situated in an area which is highly endemic for tuberculosis. Formal ethical approval was taken from the Institutional Ethics Committee, King George’s Medical University, U.P., India. Written informed consent was obtained from all patients and/or their legal guardians.

### Inclusion criteria

All consecutive newly-diagnosed patients of tuberculous meningitis, fulfilling the consensus diagnostic criteria as described by Marais et al., were included. Patients were categorized into definite, probable, or possible tuberculous meningitis groups on the basis of clinical, imaging, and laboratory criteria. The definite cases of tuberculous meningitis had either the presence of acid-fast bacilli in cerebrospinal fluid, or mycobacterium could be cultured from cerebrospinal fluid, or cerebrospinal fluid was positive for mycobacterial nucleic acids by polymerase chain reaction. The probable cases fulfilled the clinical entry criteria plus a total diagnostic score of 10 or more points (when cerebral imaging was not available) or 12 or more points (when cerebral imaging was available) plus exclusion of alternative diagnoses. The possible cases included clinical entry criteria plus a total diagnostic score of 6–9 points (when cerebral imaging was not available) or 6–11 points (when cerebral imaging was available) along with the exclusion of alternative diagnoses [9].

### Disease severity

Patients were classified as per British Medical Research Council staging system. Patients with stage-I disease had a Glasgow Coma Scale score of 15 with no focal neurologic signs; patients with stage-II had signs of meningeal irritation with slight or no alteration of sensorium and minor neurological deficit (like cranial nerve palsies) or no deficit (Glasgow coma scale score 11 to14), and patients with stage-III had severe alteration of sensorium, convulsions, focal neurological deficit and involuntary movements (Glasgow coma scale score <10) [10].

### Evaluation of patients

All enrolled patients were subjected to detailed clinical, radiological, and laboratory evaluation. Occurrence of systemic tuberculosis, such as involvement of pulmonary system, lymph node, bone, and other organs, were recorded. The work up included complete blood count, peripheral blood smear examination, erythrocyte sedimentation rate, blood urea nitrogen, blood sugar, serum creatinine, serum electrolytes, liver function tests, chest X-ray, and enzyme linked immunosorbent assay for HIV. Cerebrospinal fluid biochemical and microscopic examination, including India ink preparation, were performed. Cerebrospinal fluid sediments were stained, cultured (Lowenstein Jensen media), and tested for drug susceptibility by standard methods. Cerebrospinal fluid specimens were also tested for mycobacterial nucleic acids by polymerase chain reaction. Appropriate patients were also evaluated by performing bacterial screen with culture, cryptococcal latex antigen detection, antinuclear antibody, antineutrophil cytoplasmic antibody screen, hepatitis B and C, and angiotensin converting enzyme level. Patients were subjected to MR imaging of the brain and/or spinal cord using Signa Excite 1.5 Tesla instrument (General Electric Medical Systems, Milwaukee, WI, USA.

### Exclusion criteria

Patients with cryptococcal meningitis or any other cause for meningitis were excluded from the study.

### Definition of paradoxical reaction

A paradoxical reaction was defined as the worsening of pre-existing tuberculous lesions or the appearance of new tuberculous lesions in patients whose clinical symptoms initially improved and had been on anti-tuberculosis treatment for at least 10 days [2, 3, 11]. For the purpose of the calculation of “time of onset of paradoxical reaction” in the study participants only clinical deteriorations were considered from the time of initiation of anti-tuberculosis treatment; non-symptomatic radiological worsening was defined either at the time of clinical deteriorations or at the pre-defined follow-up visit.

### Definition of other neuroimaging findings

Hydrocephalus was defined as ventriculomegaly with Evan’s ratio (maximal width of frontal horns/maximal width of inner skull) of more than 30 % and/or size of one or both temporal horns greater than 2 mm. Communicating hydrocephalus was characterized with ventriculomegaly with a dilated fourth ventricle. Meningeal inflammation, on gadolinium-enhanced MRI, was defined as an enhancement of pia-arachnoid mater covering subarachnoid spaces of the sulci and basal cisterns. Exudates were defined as a thick area of enhancement, predominantly in basal cisterns and Sylvian fissure. Optochiasmatic tuberculoma and arachnoiditis were characterized with confluent enhancing lesions involving interpeduncular fossa, pontine cistern, and perimesencephalic and suprasellar cisterns. Spinal arachnoiditis was defined with the presence of enhancement of spinal meninges, and nerve roots, obliteration of the spinal subarachnoid space, cysts and loculations in subarachnoid space, or syrinx formation. Tuberculomas were defined as discrete or coalescing cerebral masses showing nodular or ring shaped enhancement. Infarcts were areas of abnormal signal intensity in a vascular distribution, predominantly in periventricular region. Infarcts appeared hyperintense on T2-weighted images and hypointense on T1-weighted images, with corroborating changes on diffusion-weighted and apparent diffusion coefficient images.

### Treatment

All included patients received anti-tuberculosis treatment as per World Health Organization guidelines for the treatment of central nervous system tuberculosis. Patients received 2 months of daily oral isoniazid (5 mg/ kg of body weight; maximum, 300 mg), rifampicin (10 mg/ kg; maximum, 600 mg), pyrazinamide (25 mg/kg; maximum, 2 g/day) and intramuscular streptomycin (20 mg/kg; maximum 1 gm/day), followed by at least 7 months administration of isoniazid and rifampicin [12]. Corticosteroid regimen consisted of intravenous dexamethasone for 4 weeks (0.4 mg/kg body weight per day and then tapered off decreasing 0.1 mg/kg every week) and then oral treatment for 4 weeks, starting at a total of 4 mg per day and decreasing by 1 mg each week [13]. Pyridoxine was given orally 20–40 mg/day to all the patients. In addition, patients received symptomatic treatment as well. Neurosurgical opinion was sought, whenever required.

In HIV-infected patients antiretroviral therapy was administered under supervision of the Nodal anti-retroviral therapy unit. Antiretroviral therapy was started as soon as anti-tuberculosis treatment was tolerated (between 2 weeks and 2 months), as per National AIDS Control Organization, Government of India, guidelines [14].

### Disability assessment

Disability assessment was done using Barthel index (BI), which is a 20 point scoring system. Assessment of disability included degree of dependence for bowel and bladder, grooming, toilet use, transfer, mobility, dressing, feeding, use of stairs and bathing. For each activity, a score of 0 indicated a complete dependence, and an individual score of 2 or 3 indicated that the patient was able to perform activities independently. A score of ≤ 12 indicated poor functional status and a score of >12 indicated good functional status.

### Follow up

Patients were regularly followed up for 9 months. Besides a defined follow up at 3, 6, and 9 months, clinical assessment and cerebrospinal fluid analysis were also done at the time of clinical deterioration. Changes in cerebrospinal fluid parameters (like cells, protein and sugar) were noted. Polymorphonuclear predominance was defined when these constituted >50 % of cells. Repeat neuroimaging were performed after 3 months and 6 months, or at periods of clinical deteriorations. Neuroimaging were looked for any deterioration or new findings. Assessment of disability was done at the end 9 months of follow up. Barthel index >12 was considered as good outcome. Intervening visits mandated by clinical deteriorations generated off-pre-defined visit data which was additionally recorded.

### Statistical analysis

Statistical analysis was performed using the Statistical Package for Social Sciences, version 16 for windows (SPSS, Chicago IL, USA). Both univariate and multivariate analysis were done to evaluate the predictors of paradoxical reaction. Univariate analysis was performed by Chi-square test for categorical variables and independent sample “t” test for continuous variables. Odds ratio with 95 % confidence interval were ascertained. For multivariate analysis, binary logistic regression was performed to see the impact of individual predictors of paradoxical reaction. Paired sample “t” test and McNemar tests were used to compare quantitative and qualitative variables before and during development of paradoxical reaction. Kaplan Meier analysis was performed to estimate the event free survival for the outcome with or without baseline paradoxical reaction using the Log Rank test. Statistical significance was defined at a p value of <0.05. All statistical analyses were two-tailed.

## Results

### Baseline characteristics

**Fig. 1:**
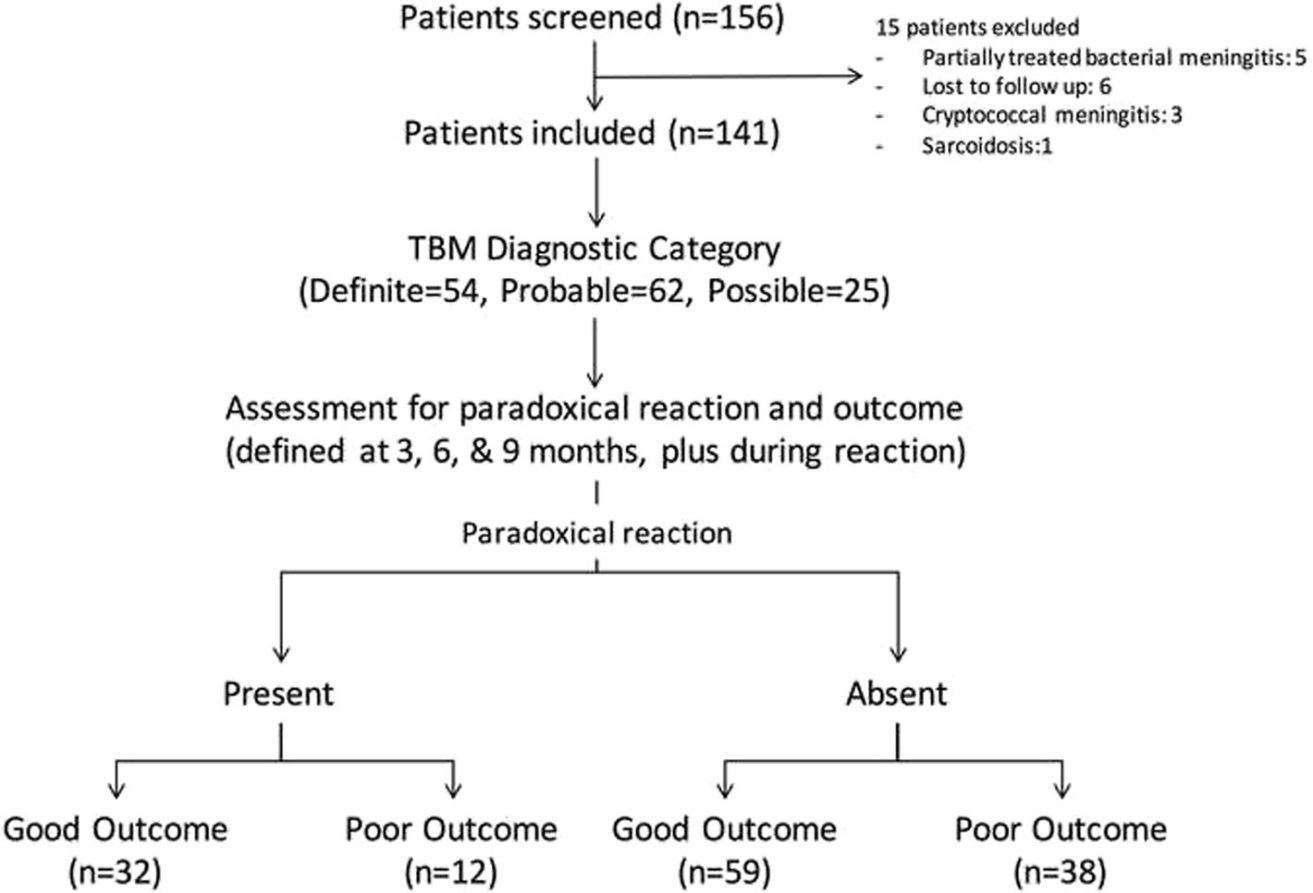
Flow chart of the study

We screened 156 consecutive patients of tuberculous meningitis during the study period which ranged from October 2013 to March 2015. Fifteen patients were excluded. Reasons for exclusion of 15 patients have been provided in Fig. 1. Finally, 141 patients were included and evaluated. Majority of our patients 66 (46.8 %), at inclusion, were in stage-II. Thirteen patients were HIV positive. There were 54 (38.3 %) microbiologically-confirmed cases. One patient had multidrug resistant tuberculous meningitis and was included in the non-paradoxical reaction group. In the paradoxical reaction group the mean duration of illness was 42.93 ± 21.58 (median = 38, range 18–107) days while in the non-paradoxical reaction group the mean duration of illness was 52.49 ± 27.54 (median = 45, range 5–180) days (Table 1).

**Table 1:** Baseline characteristics, radiological and cerebrospinal fluid parameters, and outcome of patients with tuberculous meningitis

	Total numbers of patients included (*n*=141)	Paradoxical manifestation (*n*=44)	No paradoxical manifestation (*n*=97)
Demographic			
Age (Mean±SD)	29.75±12.79	27.295±10.95	30.866±13.44
Duration of illness (Mean±SD)	49.51±26.13	42.932±21.58	52.495±27.53
Gender (n, %)			
Male	76(53.9 %)	18(40.9 %)	58(59.8 %)
Female	65(46.1 %)	26(59.1 %)	39(40.2)
HIV (n, %)	13(9.2 %)	11(25 %)	2(2.1 %)
Clinical (n, %)			
BMRC stage 1	31(22.0 %)	8(18.2 %)	23(23.7 %)
BMRC stage 2	66(46.8 %)	21(47.7 %	45(46.4 %)
BMRC stage 3	44(31.2 %)	15(34.1 %)	29(29.9 %)
Fever	138(97.8 %)	43(97.7 %)	95(97.9 %)
Headache	131(92.90 %)	39(88.6 %)	92(94.8 %)
Seizure	48(34.0 %)	12(27.3 %)	36(37.1 %)
Altered Sensorium	44(31.2 %)	15(34.1 %)	29(29.9 %)
Decreased vision	35(24.8 %)	10(22.7 %)	25(25.8 %)
Hemiparesis	23(16.3 %)	7(15.9 %)	16(16.5 %)
Paraparesis	12(8.5 %)	2(4.5 %)	10(10.3 %)
Meningeal signs	125(88.7 %)	41(93.2 %)	84(86.6 %)
Lymphadenopathy	13(9.2 %)	10(22.7 %)	3(3.1 %)
Diplopia	59(41.8 %)	17(38.6 %)	42(43.3 %)
Cranial Nerve involvement	80(56.7 %)	27(61.4 %)	53(54.6 %)
Papilloedema	50(62.5 %)	13(29.5 %)	42(43.3 %)
Imaging (n, %)			
Hydrocephalus	53(37.6 %)	13(29.5 %)	40(41.2 %)
Basal Exudates	38(27.0 %)	9(20.5 %)	29(29.9 %)
Tuberculoma	51(36.2 %)	15(34.1 %)	36(37.1 %)
Infarct	25(17.7 %)	7(15.9 %)	18(18.6 %)
Optochiasmatic arachnoiditis	21(14.9 %)	7(15.9 %)	14(14.4 %)
Spinal arachnoiditis	12(8.5 %)	2(4.5 %)	10(10.3 %)
Cerebrospinal fluid			
White blood cells (cells/mL±SD)	189.610±185.66	200.864±121.26	184.505±208.809
Protein mg%	253.704±455.84	188.5382±115.145	283.2649±542.511
Sugar mg%	35.311±18.46	33.620±18.231	36.086±18.61
Neutrophil differential in percentage, mean±SD	17.21±19.250	19.705±21.368	16.072±18.210
Lymphocyte differential in percentage (mean±SD)	83.65±18.02	80.295±21.368	85.175±16.166
AFB smear (n, %)	4(2.8 %)	1(2.27 %)	3(3.09 %)
AFB culture positive (n, %)	21(14.9 %)	11(25 %)	10(10.30 %)
TB PCR-positive (n, %)	50(35.5 %)	22(50 %)	28(28.86 %)
Diagnostic category (n, %)			
Definite	54(38.3 %)	20(45.5 %)	34(35.1 %)
Probable	62(44.0 %)	19(43.2 %)	43(44.3 %)
Possible	25(17.7 %)	5(11.4 %)	20(20.6 %
Disability (n, %)			
Baseline BI(<12)	93(66.0 %)	34(77.3 %)	59(60.8 %)
Baseline BI(>12)	48(34.0 %)	10(22.7 %)	38(39.2 %)
Outcome (n, %)			
Poor (BI<12)	50(35.5 %)	12(27.3 %)	38(39.2 %)
Good (BI>12)	91(64.5 %)	32(72.7 %)	59(60.8 %)

*AFB* acid fast bacilli, *BMRC* British Medical Research Council, *HIV* human immunodeficiency virus, *BI* Barthel index, *TB PCR* tuberculosis polymerase chain reaction

### Incidence of paradoxical reaction

In our study, 44 (31.2 %) patients developed some form of paradoxical reaction. In majority (29 patients), paradoxical reactions were noted within 3 months of start of anti-tuberculosis treatment. Mean duration of paradoxical reaction was 82.45 days (16–320 days) (Additional file file 1: Table S1).

### Manifestations of paradoxical reaction

**Fig. 2:**
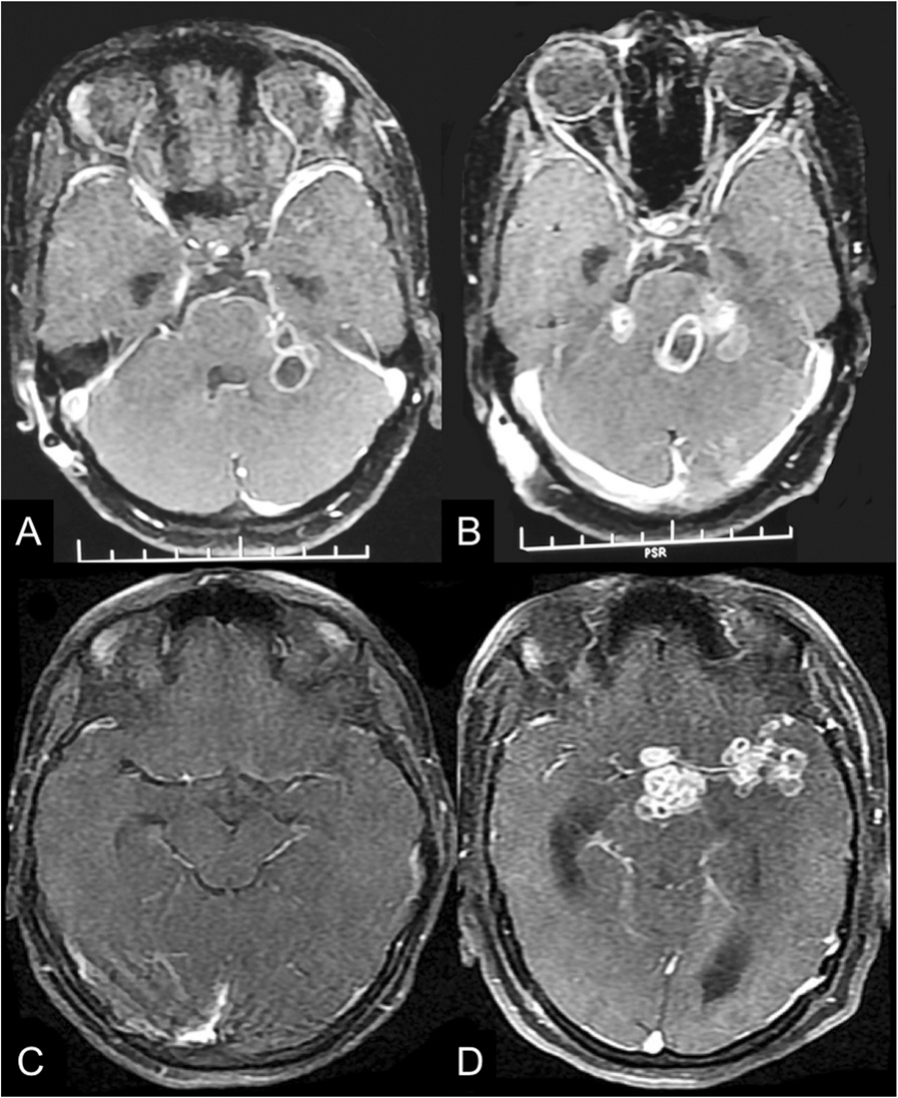
T1-weighted Gadolinium-enhanced MRI (axial sections) of the brain of a patient with TBM depict the development of additional (paradoxical) tuberculoma (Baseline-**a**, at 5th month-**b**) at the pontocerebellar level. Similar sequence in another patient shows development of paradoxical optochiasmatic arachnoiditis along with multiple tuberculomas (Baseline-**c**, at 3rd month-**d**)

Fever, headache, altered sensorium, decreased vision, and seizures were common clinical paradoxical reactions among 44 patients who presented with a paradoxical reaction. Among neuroimaging changes, enhancing basal exudates, a new or enlargement of pre-existing tuberculoma, development of infarcts, and increasing ventriculomegaly were the more frequent paradoxical reactions. Twenty-six (59 %) patients developed either a new (15 patients) or enlargement (11 patients) of pre-existing tuberculoma. Twenty patients demonstrated newly developed hydrocephalus while in 7 cases ventricular size had increased further. Optochiasmatic arachnoiditis was the other common but disabling paradoxical manifestation. Four patients, additionally, had spinal arachnoiditis. In 41 patients, cerebrospinal fluid paradoxically worsened (increase in cells with or without rise in protein level). Out of the remaining 3, two patients demonstrated a decrease in the cell count but with polymorph predominance (not present at the baseline) while in one the cerebrospinal fluid was normal. In cerebrospinal fluid, major changes observed were increase in protein level and increase in polymorphs. In 3 patients with paradoxical reaction, only cerebrospinal fluid changes (without imaging changes) were noted. Fourteen patients had paradoxical lymphadenopathy while three patients had developed miliary tuberculosis (Fig. 2) (Additional file 1: Table S1). Statistically significant changes from the baseline to the time of development of paradoxical reaction was observed in fever, headache, hydrocephalus, tuberculoma, c-reactive protein, erythrocyte sedimentation rate, and cerebrospinal fluid polymorphs, lymphocytes, and protein (Table 2).

**Table 2:** Comparative analysis of variables of patients with paradoxical reaction

Variable	At baseline	During pxaradoxical reaction	*P* value
Fever	43	29	*.001*
Headache	39	27	*.008*
Seizure	12	11	1.0
Altered Sensorium	15	12	.664
Decreased vision	10	16	.180
Hemiparesis	7	9	.791
Paraparesis	2	4	.688
Hydrocephalus	13	27	*.009*
Tuberculoma	15	26	*.019*
Basal Exudate	9	12	.648
Optochiasmatic arachnoiditis	7	12	.332
Infarct	7	9	.791
Spinal arachnoiditis	2	4	.688
CRP	19	28	*.004*
ESR	27.818±4.876	33.20±5.889	*.027*
CSF cells	200.864±121.260	210.295±125.761	.058
CSF Polymorph	19.705±21.368	37.84±23.716	*.008*
CSF Lymphocytes	80.295±21.368	62.39±23.398	*.012*
CSF Protein	188.538±115.145	261.355±152.0650	*<.001*
CSF sugar	33.620±43.2566	43.256±17.998	.261

*CRP* C-reactive protein, *CSF* cerebrospinal fluid, *ESR* Erythrocyte sedimentation rate

Note: Statistically significant *P*-values are depicted in italics

### Predictors of paradoxical reaction

On univariate analysis, female gender (*p* = 0.037), HIV positivity (*p* = <0.001), lymphadenopathy (*p* = 0.001) and a shorter duration of illness (*p* = 0.028) were significantly associated with development of paradoxical reaction (Table 3). On multivariate analysis, female gender (*p* = 0.013, OR =2.87, CI 1.24-6.62), HIV positivity (*p* = 0.010, OR = 31.49; CI = 2.31-426.21), and a shorter duration of illness (*p* = 0.049, OR = 0.98 CI = .963-1.00) were found to be statistically significant predictors of paradoxical reaction (Table 4).

**Table 3:** Predictors of paradoxical reaction on univariate analysis

Characteristic	Paradoxical reaction		*P* value	Odds ratio	95 % Confidence interval	
	Present (*n*=44)	Absent (*n*=97)			Lower bound	Upper bound
Age in years Mean ± SD	27.295 ± 10.958	30.866 ± 13.446	0.125		−1.004	8.145
Duration of illness (days) Mean ± SD	42.932± 21.583	52.495± 27.538	*0.028*		0.276	18.850
Gender (female)	26 (59.1 %)	39 (40.2 %)	*0.037*	2.148	1.04	4.436
HIV positive	11(25 %)	2(2.1 %)	*<0.001*	15.833	3.334	75.184
Fever	43(97.7 %)	95(97.9 %)	0.936	0.905	0.080	10.255
Headache	39(88.6 %)	92(94.8 %)	0.183	0.424	0.116	1.548
Seizure	12(27.3 %)	36(37.1 %)	0.253	0.635	0.291	1.387
Altered Sensorium	15(34.1 %)	29(29.9 %)	0.618	1.213	0.567	2.593
Decreased vision	10(22.7 %)	25(25.8 %)	0.698	0.847	0.366	1.960
Hemiparesis	7(15.9 %)	16(16.5 %)	0.930	0.958	0.363	2.526
Paraparesis	2(4.5 %)	10(10.3 %)	0.341	0.414	0.087	1.976
Meningeal signs	41(93.2 %)	84(86.6 %)	0.253	2.115	0.571	7.837
Lymphadenopathy	10(22.7 %)	3(3.1 %)	*0.001*	9.216	2.393	35.496
Diplopia	17(38.6 %)	42(43.3 %)	0.603	0.825	0.398	1.707
Cranial Nerve involvement	27(61.4 %)	53(54.6 %)	0.455	1.319	0.638	2.727
Papilloedema	13(29.5 %)	42(43.3 %)	0.121	0.549	0.256	1.177
Hydrocephalus	13(29.5 %)	40(41.2 %)	0.184	0.598	0.279	1.282
Basal Exudates	9(20.5 %)	29(29.9 %)	0.242	0.603	0.257	1.413
Tuberculoma	15(34.1 %)	36(37.1 %)	0.729	0.876	0.415	1.850
Infarct	7(15.9 %)	18(18.6 %)	0.703	0.830	0.319	2.161
Optochiasmatic Arachnoiditis	7(15.9 %)	14(14.4 %)	0.820	1.122	0.418	3.008
Spinal cord	2(4.5 %)	10(10.3 %)	0.256	0.414	0.087	1.976
CRP	19(43.2 %)	38(39.2 %)	0.653	1.180	0.573	2.430
Stage 3 TBM	15(34.1 %)	29(29.9 %)	0.618	1.213	0.567	2.593
Baseline BI (<12)	34(77.3 %)	59(60.8 %)	0.056	2.190	0.970	4.944
Definite TBM	20(45.5 %)	34(35.1 %)	0.312	1.544	.748	3.189
Good outcome (BI>12)	32(72.7 %)	59(64.8 %)	0.171	0.582	0.267	1.268
ESR mm in 1 h Mean ± SD	27.818 ± 4.876	27.093 ± 7.593	0.497		−3.194	1.743
CSF Cells/μL Mean ± SD	200.864 ± 121.261	184.505 ± 208.810	0.630		83.265	56.548
CSF protein (mg/dl) Mean ± SD	188.5382 ± 115.145	283.2649 ± 542.511	0.254		−68.924	258.377
CSF sugar (mg/dl) Mean ± SD	33.620 ± 18.231	36.086 ± 18.617	0.465		4.193	9.125

*BI* Barthel Index, *CRP* C-reactive protein, *CSF* cerebrospinal fluid, *ESR* Erythrocyte sedimentation rate, *HIV* Human Immunodeficiency Virus, *TBM* Tuberculous meningitis

Note: Statistically significant *P*-values are depicted in italics

**Table 4:** Predictors of paradoxical reaction on multivariate analysis

Characteristic	*P* value	Odds ratio	95 % Confidence interval	
			Lower bound	Upper bound
Duration	*.049*	.981	0.963	1.000
Gender (female)	*.013*	2.873	1.246	6.626
HIV	*.010*	31.407	2.314	426.215
Lymphadenopathy	.813	0.746	0.066	8.465

*HIV* human immunodeficiency virus

Note: Statistically significant *P*-values are depicted in italics

### Impact on prognosis

**Fig. 3:**
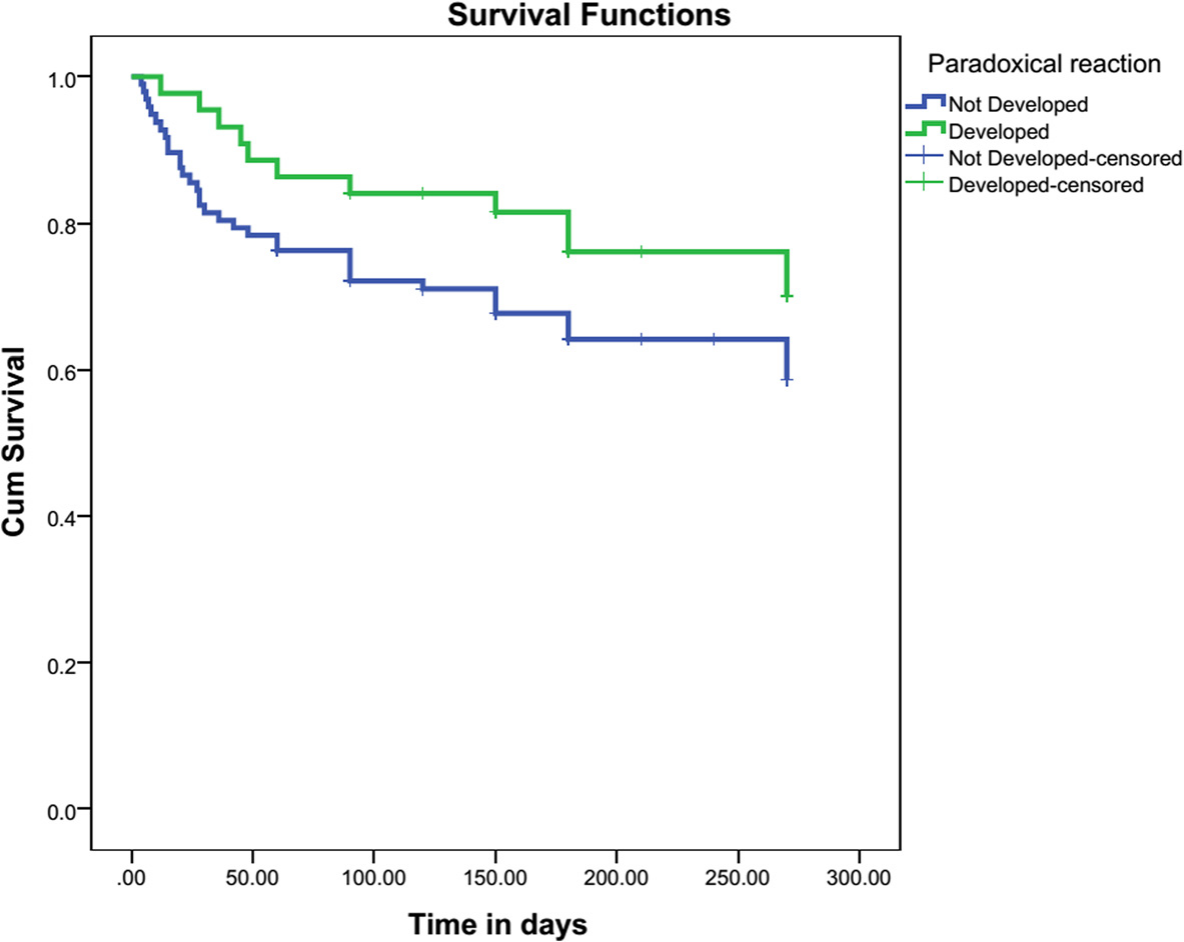
Kaplan Meyer survival analysis showed no significant difference in the cumulative survival of patients with or without paradoxical reaction (Log rank test: *X* ^2^ (1) = 2.198, *P* = 0.138)

Overall, duration of illness, alteration in consciousness, stage-III of tuberculous meningitis, and hydrocephalus predicted poor prognosis among patients with tuberculous meningitis. In the paradoxical group 24 % patients had poor outcome compared to 35.2 % with good outcome, which was statistically not significant (Table 5). We did not observe any significant difference in the proportion of patients having poor disability status after end of follow up. Kaplan-Meier survival statistics did not demonstrate any significant difference in survival between both groups (Fig. 3).

**Table 5:** Predictors of disability and outcome on univariate analysis in patients with tuberculous meningitis

Characteristics	BI≤12 (*n*=50)	BI>12 (*n*=91)	*P* value	Odds Ratio	95 % Confidence interval	
					Lower bound	Upper bound
Age	29.320±11.881	29.989±13.322	.768		−3.797	5.135
Duration of illness	42.140±21.204	53.560±27.761	*.013*		2.495	20.345
Gender	22(44 %)	54(59.3 %)	.080	.538	.268	1.082
HIV						
Fever	49(98 %)	89(97.8 %)	.938	1.101	.097	12.453
Headache	44(88 %)	87(95.6 %)	.092	.337	.090	1.257
Seizure	15(30.0 %)	33(36.3 %)	.453	.753	.359	1.580
Altered Sensorium	31(62.0 %)	13(14.3 %)	*<.001*	9.789	4.316	22.206
Decreased vision	16(32.0 %)	19(20.9 %)	.144	1.783	.817	3.891
Hemiparesis	9(18.0 %)	14(15.4 %)	.688	1.207	.482	3.027
Paraparesis	6(12.0 %)	6(6.6 %)	.271	1.932	.588	6.342
Meningeal signs	44(88.0 %)	81(89 %)	.856	.905	.308	2.657
Lymphadenopathy	4(8.0 %)	9(9.9 %)	.711	.792	.231	2.716
Diplopia	19(38.0 %)	40(44.0 %)	.493	.821	.402	1.675
Cranial Nerve involvement	28(56.0 %)	52(57.1 %)	.896	.955	.476	1.914
Papilloedema	18(36.0 %)	37(40.7 %)	.587	.821	.402	1.675
Hydrocephalus	40(80.0 %)	13(14.3 %)	*<.001*	24.0	9.677	59.520
Basal Exudates	12(24.0 %)	26(28.6 %)	.558	.789	.357	1.744
Tuberculoma	22(44.0 %)	29(31.9 %)	.151	1.680	.825	3.422
Infarct	9(18.0 %)	16(17.6 %)	.950	1.029	.418	2.533
Optochiasmatic arachnoiditis	7(14.0 %)	14(15.4 %)	.825	.895	.336	2.388
Spinal arachnoiditis	6(12.0 %)	6(6.6 %)	.271	1.932	.588	6.342
Stage-III TBM	31(62.0 %)	13(14.3 %	*<.001*	9.789	4.316	22.206
Definite TBM	23(46.0 %)	31(34.1 %)	.163			
Paradoxical reaction	12(24.0 %)	32(35.2 %)	.171	.582	.267	1.268
CSF Cells	159.340	206.242	.152		−17.474	111.277
CSF protein	256.772	252.019	.953		−163.99	154.488
CSF sugar	35.112	35.422	.925		−6.153	6.773

*CSF* cerebrospinal fluid, *HIV* human immunodeficiency virus, *BI* Barthel index, *TBM* tuberculous meningitis

Note: Statistically significant *P*-values are depicted in italics

## Discussion

In this prospective study approximately one-third of patients with tuberculous meningitis developed paradoxical changes. In almost all patients, clinical/imaging paradoxical manifestations accompanied paradoxical cerebrospinal fluid changes. We encountered a variety of manifestations of paradoxical reactions. These paradoxical clinical and neuroimaging manifestations were either aggravated or appeared for the first time during illness, despite of an adequate anti-tuberculosis treatment. Expansion of existing cerebral tuberculomas and appearance of new tuberculomas, hydrocephalus, and optochiasmatic and spinal arachnoiditis, were common paradoxical manifestations.

Paradoxical reactions in these patients result because of an excessive inflammatory response against mycobacterial antigens. Mycobacterial cell wall antigens are present in the affected brain tissues and trigger an exaggerated inflammatory reaction following treatment with anti-tuberculosis drugs. Cheng and colleagues had noted a surge in blood lymphocyte counts accompanied by an increased tuberculin skin reaction during paradoxical response indicating that this immune response is similar to immune restoration syndrome seen in HIV-infected patients. Possibly, due to a paradoxical shift in the cellular components of the cerebrospinal fluid (from lymphocytic predominance to polymorph predominance), neutrophils also play a role in the pathogenesis of paradoxical reaction in patients with tuberculous meningitis. [15] Sütlaş and co-workers followed 61 patients with confirmed or presumed tuberculous meningitis. Patients were categorized into two groups according to the presence of paradoxical response (progressive increase of lymphocytes or increase of polymorphonuclear cells instead of lymphocytes) in cerebrospinal fluid samples. Paradoxical response in the cerebrospinal fluid was seen in 20 patients. New tuberculomas developed more frequently in the group having paradoxical cerebrospinal fluid response. We had similar observations in our study where 41 patients with clinical/imaging paradoxical changes had paradoxical cerebrospinal fluid changes. Interestingly, in two patients where the cells actually decreased, there was a shift towards polymorph predominance. It has been observed that paradoxical cerebrospinal fluid changes occur after several weeks of starting anti-tuberculosis treatment and may not be associated with any clinical or neuroimaging deterioration [16].

We observed that female gender, concomitant HIV infection, and a shorter duration of illness, were significant predictors of paradoxical reactions in patients with tuberculous meningitis. Females exhibit more-robust immune responses to antigenic challenges, like infection and vaccination, in comparison to that in males. Given the fact that immune cells express specific receptors for sex hormones and are responsive to changes in hormone levels, this gender variation might be explained by the modulatory effect of female sex hormones on the immune response towards *Mycobacterium tuberculosis* associated antigens [17, 18].

Patients with disseminated or extrapulmonary disease are considered to have higher bacillary/antigen loads, with a relative immunodeficient response, resulting in an increased risk of both paradoxical reactions and IRIS. Use of bactericidal drugs, such as isoniazid, may cause a massive release of bacterial components and provoke inflammatory changes that could produce clinical deterioration and imaging changes [19]. Another significant predictor of paradoxical reaction was a shorter duration of illness. Possibly, a large mycobacterial load and subsequent release of mycobacterial antigen following anti-tuberculosis treatment resulted in a higher frequency of paradoxical reactions among patients who developed tuberculous meningitis comparatively rapidly.

A substantial proportion of our HIV-infected patients (11 out of 13 patients) developed paradoxical reactions. Many studies in the past have noted that many patients develop tuberculous meningitis associated IRIS. Manifestations of central nervous system tuberculosis IRIS include meningitis, cerebral parenchymal tuberculoma, brain abscesses, spinal cord complications like myeloradiculopathy, and spinal epidural abscesses [20–22]. In a prospective study, 47 % (16/34) of tuberculous meningitis patients developed tuberculous meningitis-IRIS. At inclusion, tuberculous meningitis-IRIS patients had a higher cerebrospinal fluid neutrophil counts compared with non-tuberculous meningitis-IRIS patients [20]. There is a consensus that antiretroviral therapy should be started early (around 2 weeks) in HIV/tuberculosis co-infected patients. In HIV and tuberculous meningitis co-infected patients, because of a potential risk of tuberculous meningitis associated IRIS, antiretroviral therapy may be delayed; however, there is no consensus on this point [23].

We observed that the development of paradoxical reactions did not adversely affect the outcome of tuberculous meningitis. Several studies in the past had similar observations. Clinical, laboratory, radiological, and outcome data of 14 cases of paradoxical tuberculoma in tuberculous meningitis patients (out of 22 patients who had intracranial tuberculoma) were compared with 41 tuberculous meningitis patients without tuberculomas. The analysis suggested that there was no statistical difference between two groups regarding age, stage of tuberculous meningitis, duration between disease onset and treatment initiation, cerebrospinal fluid protein levels, and in long-term disabilities [24]. Many manifestations of paradoxical reactions like optochiasmatic and spinal arachnoiditis, and a large cerebral tuberculoma are serious conditions associated with severe disabilities, and a potential for death [25]. These conditions warrant urgent treatment with immunomodulatory drugs. High doses of corticosteroids are currently the most preferred treatment. Many other immunomodulatory drugs like, tissue necrosis factor-α antagonists, thalidomide, and interferon– γ have also been used, though sparingly [3].

There were certain limitations of our study. It is difficult to pin-point exactly the timing of onset of a paradoxical reaction clinically. Besides this, the differential diagnosis of a paradoxical reaction may also pose a challenge. A wrong diagnosis, possibility of treatment failure, multidrug-resistance, atypical mycobacterial infection, drug toxicity, or clinical deterioration due to some unrelated cause should always be co-considered. One of our patients with apparent paradoxical reaction later turned out to have multidrug-resistant tuberculosis.

## Conclusion

Paradoxical reaction occurs in approximately one-third of patients with tuberculous meningitis. Female gender, concomitant HIV infection, and a shorter duration of illness are significant predictors of such reactions. Duration of illness, alteration in consciousness, stage-III of tuberculous meningitis, and hydrocephalus, predict a poor prognosis in patients with tuberculous meningitis; paradoxical reactions, however, do not seem to affect the outcome adversely.

## Abbreviations

AIDS, acquired immune deficiency syndrome; BI, barthel index; HIV, human immunodeficiency virus; IRIS, immune reconstitution inflammatory syndrome; MRI, magnetic resonance imaging

## Additional file

Additional file 1: Table S1. Clinicoradiological, biochemical and outcome details of patients with paradoxical reaction in tuberculous meningitis. (DOCX 30 kb)
