# Supplementary material for: Paradoxical reaction in tuberculous meningitis: presentation, predictors and impact on prognosis
Source: BMC Infect Dis. 2016 Jun 21;16:306. doi: 10.1186/s12879-016-1625-9 (PMC4915108; doi:10.1186/s12879-016-1625-9)
Supplement: Additional file 1: Table S1. — Clinicoradiological, biochemical and outcome details of patients with paradoxical reaction in tuberculous meningitis. (DOCX 30 kb) [file 12879_2016_1625_MOESM1_ESM.docx]

**Supplementary Table: Clinicoradiological, biochemical and outcome details of patients with paradoxical reaction in tuberculous meningitis**

| **S. No** | **Age group*/ Gender** | **Duration of illness (days)** | **Stage of TBM** | **HIV status** | **TBM**  **Diagnostic Category** | **Manifestations of Paradoxical reaction** | | | | | | | **MBI** | | **Time to onset of paradoxical reaction^#^** | **Outcome^$$^** |
| --- | --- | --- | --- | --- | --- | --- | --- | --- | --- | --- | --- | --- | --- | --- | --- | --- |
|  |  |  |  |  |  | **Extra-CNS manifestations** | | | **CNS manifestations** | | | |  |  |  |  |
|  |  |  |  |  |  | **Lymphadenopathy** | **PTB** | **Miliary tuberculosis^$^** | **Clinical** | **Neuroimaging** | **CSF** | | **Baseline** | **At 9 months** |  |  |
|  |  |  |  |  |  |  |  |  |  |  | **Cells** | **Protein** |  |  |  |  |
| 1 | III/F | 32 | I | Negative | Definite | Absent | Absent | Absent | Hemiparesis, 6^th^ cranial palsy | Tuberculoma, basal exudates, hydrocephalus, infarct | Cells increase, polymorph predominance | Increased | 16 | 20 | 27** | Good |
| 2 | III/M | 44 | II | Negative | Possible | Absent | Absent | Absent | Decreased vision, Altered sensorium, paraparesis | Optochiasmatic arachnoiditis, spinal arachnoiditis, | Cells increase, lymphocyte predominance | Increased | 12 | 18 | 29** | Good |
| 3 | III/F | 29 | I | Negative | Probable | Absent | Present | Absent | Decreased vision, 6^th^ cranial palsy, seizures | Tuberculoma, basal exudates | NL | Increased | 4 | 10 | 320 | Death |
| 4 | IV/M | 32 | II | Negative | Probable | Absent | Absent | Absent | Decreased vision, hemiparesis | Hydrocephalus, optochiasmatic arachnoiditis, infarct | Cells increase, lymphocyte predominance | Increased | 12 | 18 | 101 | Good |
| 5 | V/F | 18 | II | Negative | Definite | Absent | Absent | Absent | Seizures, altered sensorium | Tuberculoma | Cells increase, polymorph predominance | Increased | 14 | 20 | 139 | Good |
| 6 | II/F | 107 | I | Negative | Definite | Present | Absent | Absent | Paraparesis | Hydrocephalus, spinal arachnoiditis | Cells increase, lymphocyte predominance | Increased | 16 | 20 | 16** | Good |
| 7 | V/F | 38 | II | Negative | Probable | Present | Absent | Absent | 3^rd^ cranial nerve palsy, Decreased vision | Tuberculoma, basal exudates | Cells increase, lymphocyte predominance | Increased | 12 | 19 | 59 | Good |
| 8 | III/M | 31 | III | Negative | Possible | Absent | Absent | Absent | 3^rd^ and 6^th^ cranial nerve palsy, hemiparesis | Infarct, basal exudates | Cells increase, polymorph predominance | NL | 6 | 11 | 27** | Poor |
| 9 | II/M | 76 | II | Positive | Definite | Present | Absent | Absent | 6^th^ cranial nerve palsy | Hydrocephalus, basal exudates | Cells increase, polymorph predominance^##^ | NL | 12 | 18 | 71 | Good |
| 10 | IV/F | 91 | II | Negative | Definite | Absent | Absent | Absent | Decreased vision, altered sensorium | Hydrocephalus, optochiasmatic arachnoiditis | Cells increase, lymphocyte predominance | NL | 6 | 10 | 32 | Poor |
| 11 | V/M | 102 | II | Positive | Definite | Present | Present | Absent | Decreased vision, Headache | Tuberculoma, optochiasmatic arachnoiditis | Cells increase, lymphocyte predominance | Increased | 12 | 18 | 141 | Good |
| 12 | III/F | 71 | II | Negative | Probable | Absent | Absent | Absent | Seizures | Infarct | NL | Increased | 6 | 10 | 220 | Poor |
| 13 | II/M | 31 | III | Negative | Possible | Present | Absent | Absent | 3rd cranial nerve palsy | Tuberculoma | Cells increase, lymphocyte predominance | Increased | 18 | 20 | 154 | Good |
| 14 | III/F | 39 | I | Negative | Probable | Absent | Absent | Absent | 6th cranial nerve palsy, paraparesis | Basal exudates, hydrocephalus, spinal arachnoiditis | Cells increase, polymorph predominance | NL | 12 | 18 | 129 | Good |
| 15 | II/F | 53 | III | Positive | Probable | Absent | Absent | Absent | Altered sensorium | Tuberculoma, hydrocephalus | Cells increase, polymorph predominance | Increased | 10 | 16 | 113 | Good |
| 16 | III/F | 49 | II | Negative | Possible | Absent | Absent | Absent | Seizures | Hydrocephalus | Cells increase, lymphocyte predominance | Increased | 12 | 18 | 92 | Good |
| 17 | IV/M | 27 | I | Negative | Definite | Present | Present | Absent | Fever, Headache | Tuberculoma, hydrocephalus | Cells increase, lymphocyte predominance | Increased | 8 | 10 | 73 | Poor |
| 18 | IV/M | 101 | I | Negative | Probable | Absent | Absent | Absent | Decreased vision, 6^th^ cranial nerve palsy | Tuberculoma, basal exudates, optochiasmatic arachnoiditis, hydrocephalus, infarct | Cells increase, polymorph predominance |  | 18 | 20 | 61 | Good |
| 19 | III/F | 28 | II | Negative | Probable | Absent | Absent | Absent | Altered sensorium | Hydrocephalus | NL | NL | 5 | 14 | 52 | Good |
| 20 | III/M | 58 | II | Negative | Probable | Present | Present | Absent | Fever, Vomiting | Tuberculoma | NL | Increased | 18 | 20 | 45 | Good |
| 21 | IV/F | 25 | II | Negative | Probable | Present | Absent | Absent | Decreased vision, 3^rd^ cranial nerve palsy, Seizures | Tuberculoma, optochiasmatic arachnoiditis | NL | Increased | 6 | 10 | 42 | Poor |
| 22 | IV/F | 27 | III | Negative | Definite | Present | Absent | Present | 6th cranial nerve palsy | Basal exudates | Cells increase, polymorph predominance | NL | 12 | 18 | 39 | Good |
| 23 | II/M | 28 | II | Positive | Probable | Absent | Absent | Absent | Altered sensorium | Basal exudates | Cells increase, polymorph predominance | NL | 18 | 20 | 35 | Good |
| 24 | III/M | 34 | II | Positive | Definite | Present | Absent | Absent | Fever, Headache | Tuberculoma, hydrocephalus, infarct | NL | Increased | 12 | 20 | 32 | Good |
| 25 | II/F | 30 | I | Negative | Definite | Absent | Absent | Absent | Decreased vision | Tuberculoma, optochiasmatic arachnoiditis | Cells decrease, polymorph predominance | Increased | 6 | 0 | 27** | Death |
| 26 | III/F | 19 | III | Negative | Probable | Absent | Absent | Absent | Headache | Tuberculoma |  | Increased | 9 | 15 | 97 | Good |
| 27 | II/F | 35 | III | Negative | Probable | Present | Absent | Absent | 3rd cranial nerve palsy, altered sensorium Seizures | Tuberculoma, basal exudates, hydrocephalus, | Cells increase, polymorph predominance | NL | 6 | 10 | 130 | Poor |
| 28 | II/F | 39 | III | Positive | Definite | Absent | Present | Absent | Decreased vision, Headache | Tuberculoma, hydrocephalus, optochiasmatic arachnoiditis | Cells increase, lymphocyte predominance | NL | 10 | 18 | 89 | Good |
| 29 | III/M | 32 | III | Negative | Probable | Absent | Absent | Absent | Fever | Tuberculoma, hydrocephalus | ^##^ | Increased | 7 | 16 | 64 | Good |
| 30 | III/M | 38 | III | Negative | Definite | Absent | Absent | Present | Fever | Tuberculoma, hydrocephalus, infarct | Cells increase, polymorph predominance, | NL | 4 | 10 | 151 | Poor |
| 31 | II/F | 29 | III | Negative | Probable | Absent | Absent | Absent | Decreased vision, altered sensorium | Tuberculoma, hydrocephalus, optochiasmatic arachnoiditis | Cells decrease, polymorph predominance^##^ | Increased | 4 | 0 | 105 | Poor |
| 32 | II/M | 39 | III | Positive | Possible | Absent | Absent | Absent | Decreased vision | Tuberculoma, hydrocephalus, optochiasmatic arachnoiditis | NL | Increased | 16 | 18 | 182 | Good |
| 33 | III/M | 38 | III | Positive | Definite | Absent | Absent | Absent | Decreased vision, Seizures | Tuberculoma | Cells increase, polymorph predominance | Increased | 8 | 15 | 121 | Good |
| 34 | III/F | 29 | II | Negative | Probable | Absent | Absent | Absent | Decreased vision, Altered sensorium | Optochiasmatic arachnoiditis | NL | Increased | 5 | 13 | 108 | Good |
| 35 | I/M | 46 | I | Positive | Probable | Absent | Absent | Absent | Seizures, Headache | Tuberculoma, hydrocephalus, infarct | Cells increase, lymphocyte predominance | Increased | 20 | 20 | 83 | Good |
| 36 | III/F | 36 | III | Negative | Definite | Present | Absent | Absent | Fever, Headache | NL | Cells decrease, polymorph predominance | NL | 8 | 18 | 71 | Good |
| 37 | III/F | 41 | II | Negative | Definite | Absent | Absent | Absent | Seizures, Altered sensorium, paraparesis | Tuberculoma, basal exudates, spinal arachnoiditis, hydrocephalus | Cells increase, lymphocyte predominance | Increased | 10 | 16 | 61 | Good |
| 38 | IV/F | 32 | II | Negative | Definite | Present | Absent | Absent | Fever, headache | NL | Cells decrease, polymorph predominance^##^ | Increased | 12 | 16 | 29** | Good |
| 39 | IV/M | 38 | III | Negative | Probable | Absent | Present | Absent | Decreased vision | Hydrocephalus, optochiasmatic arachnoiditis | NL | Increased | 8 | 11 | 34 | Poor |
| 40 | III/F | 45 | II | Negative | Definite | Absent | Absent | Absent | Decreased vision, Seizures, Hemiparesis | Tuberculoma, hydrocephalus | Cells decrease, polymorph predominance | NL | 12 | 18 | 37 | Good |
| 41 | VI/M | 39 | II | Positive | Possible | Present | Absent | Present | Altered sensorium | NL | Cells increase, lymphocyte predominance | Increased | 6 | 14 | 42 | Good |
| 42 | III/F | 31 | II | Positive | Definite | Absent | Absent | Absent | Fever, Headache, Vomiting | Hydrocephalus | NL | Increased | 12 | 18 | 44 | Good |
| 43 | III/F | 39 | II | Negative | Probable | Absent | Absent | Absent | Seizures, Altered sensorium | Tuberculoma | Cells increase, polymorph predominance^##^ | Increased | 16 | 20 | 51 | Good |
| 44 | II/F | 43 | III | Negative | Definite | Absent | Absent | Absent | 6^th^ nerve palsy | Tuberculoma, basal exudates, hydrocephalus | Cells increase, lymphocyte predominance | NL | 3 | 0 | 53 | Death |

**ATT=antituberculosis treatment; CNS=Central Nervous System, CSF=Cerebrospinal fluid, HIV=Human Immunodeficiency Virus, MBI=modified Barthel index; NL=Normal limits; PTB=Pulmonary tuberculosis; TBM=tuberculous meningitis**

**# Calculated in days from the date of initiation of ATT to time of presentation with clinical manifestations**

**## Patients with baseline polymorph predominance**

**$ Excluding PTB**

**$$ All patients received ATT with corticosteroids; Good outcome was defined as MBI >12**

*** Age Group (years): I=1-10; II=11-20; III=21-30; IV=31-40; V=41-50; VI=51-60**

** **Patients developing paradoxical reaction within 1 month of initiation of ATT**
